# Evaluation of Commercial Rapid Lateral Flow Tests, Alone or in Combination, for SARS-CoV-2 Antibody Testing

**DOI:** 10.4269/ajtmh.20-1390

**Published:** 2021-06-28

**Authors:** Peter U. Fischer, Kerstin Fischer, Kurt C. Curtis, Yuefang Huang, Nicole Fetcho, Charles W. Goss, Gary J. Weil

**Affiliations:** 1 Infectious Diseases Division, Department of Medicine, Washington University School of Medicine, St. Louis, Missouri;; 2 Division of Biostatistics, Washington University School of Medicine, St. Louis, Missouri

## Abstract

Antibody tests can be tools for detecting current or past severe acute respiratory syndrome coronavirus 2 (SARS-CoV-2 [coronavirus disease 2019 (COVID-19)]) infections. Independent test evaluations are needed to document the performance with different sample sets. We evaluated six lateral flow assays (LFAs) and two laboratory-based tests (EUROIMMUN-SARS-CoV-2 ELISA and Abbott-Architect-SARS-CoV-2-IgG). We tested 210 plasma samples from 89 patients diagnosed with acute COVID-19. These samples were collected at different time points after the onset of symptoms. In addition, 80 convalescent plasma samples, and 168 pre-pandemic samples collected from adults in the United States and in Africa were tested. LFA performance varied widely, and some tests with high sensitivity had low specificity. LFA sensitivities were low (18.8–40.6%) for samples collected 0 to 3 days after symptom onset, and were greater (80.3–96.4%) for samples collected > 14 days after symptom onset. These results are similar to those obtained by ELISA (15.6% and 89.1%) and chemiluminescent microparticle assay (21.4% and 93.1%). The range of test specificity was between 82.7% and 97%. The combined use of two LFAs can increase specificity to more than 99% without a major loss of sensitivity. Because of suboptimal sensitivity with early COVID-19 samples and background reactivity with some pre-pandemic samples, none of the evaluated tests alone is reliable enough for definitive diagnosis of COVID-19 infection. However, antibody testing may be useful for assessing the status of the epidemic or vaccination campaign. Some of the LFAs had sensitivities and specificities that were comparable to those of more expensive laboratory tests, and these may be useful for seroprevalence surveys in resource-limited settings.

## INTRODUCTION

As of October 13, 2020 about 37.9 million severe acute respiratory syndrome coronavirus 2 (SARS-CoV-2) infections (coronavirus disease 2019 [COVID-19]) and 1.01 million deaths have been recorded worldwide.^1^ Reverse transcription–polymerase chain reaction (RT-PCR) tests are currently the gold standard for diagnosis of COVID-19.^2^^,^^3^ Because these assays detect viral RNA, they indicate active infection and potential infectivity. However, molecular tests are expensive, they require significant laboratory infrastructure, and they are in short supply. As the infection progresses, viral titers in the upper respiratory tract often decrease so that viral RNA may be undetectable in respiratory specimens.^4^

Antibody tests for COVID-19 detect human antibodies to viral proteins. Thus, antibody tests may be useful for the diagnosis of recent infections after antibodies have been produced or for verifying past infections in persons who were not tested by RT-PCR when they were ill; they may also have value as markers for immunity to the virus. Potential use cases for COVID-19 antibody testing have been reviewed previously.^5^ It is clear that antibody test results alone for individual patients do not provide diagnostic certainty, because no test has 100% sensitivity and specificity. However, together with the medical history and clinical signs, antibody tests results can also be very helpful for individual diagnosis. Similarly, although positive antibody test results do not guarantee immunity, they suggest prior infection and immunity. Despite these limitations, antibody tests can be useful tools for assessing COVID-19 activity in communities, and they may have some value for individual diagnosis. This is especially true in low- and middle-income countries that lack resources for widespread molecular testing. Another advantage of antibody tests over laboratory-based molecular diagnostic tests is that they do not require biosafety 3 level laboratory containment.^6^

Antibody tests for COVID-19 use a variety of test platforms, but they use a limited number of viral antigens. Most tests detect antibodies to SARS-CoV-2 spike (full length, receptor binding domain, variable domain of the spike protein) or nucleocapsid proteins, alone or in combination.^7^^–^^12^ Some tests detect IgG antibodies only, whereas others detect IgG, IgA, and/or IgM. Even when tests use the same viral antigen target, test performance can vary based on the expression system used, antigen purity, secondary antibodies, diagnostic platform, and quality control. In recent months, more than 150 rapid format antibody tests have been developed and marketed.^13^ Only a few have been evaluated independently to date, and only a small number have received emergency use authorization from the U.S. Food and Drug Administration^14^ or other authorities. Although several test evaluation studies have recently been published or posted online, most compare a limited number of antibody tests with a relatively small panel of samples from a single geographic area.^11^^,^^15^^–^^17^ Therefore, more independent test evaluation data from various geographic regions are urgently needed.

Our study focused on the evaluation of rapid, point-of-care tests using lateral flow assay (LFA) technology that could be especially helpful for antibody testing in low- and middle-income countries. We tested a panel of plasma samples that included not only confirmed COVID-19 cases from the United States, but also pre-COVID-19 samples from the United States and sub-Saharan Africa for broader specificity testing. The primary objectives of the study were to assess the sensitivity and specificity of tests for antibodies to SARS-CoV-2 proteins, and the kinetics of antibody responses relative to the time of symptom onset. Furthermore, we compared the background reactivity (false-positive rates) for sera from the United States and sera from areas in Africa where people are exposed to diseases that are absent or uncommon in the United States.

## METHODS

### Ethical approval and patient samples.

The study was approved by the Human Research Protection Office (HRPO) at Washington University (institutional review board identification no. 202004088). We tested de-identified plasma samples that were collected with informed consent under protocol HRPO 2020003085 and archived serum/plasma samples that were collected pre-COVID-19 under HRPO 201102546. The study is registered at ClinicalTrials.gov with identifier no. NC04360954.

Plasma samples from subjects with confirmed, symptomatic COVID-19 infections (WU-350) were collected at Barnes-Jewish Hospital, an affiliated teaching hospital of Washington University School of Medicine. Metadata associated with these deidentified samples included the date of sample collection, the type and date of onset of symptoms, gender, age, and other demographic information. The date of symptom onset was considered to be day 0. Convalescent plasma samples (WU-353) were collected from patients in the St. Louis area with previously documented COVID-19 infections who were now at least 14 days after symptom resolution. In this group, the earliest collection time was 21 days and the latest was 56 days after symptom onset. Initial molecular testing for these patients was performed with commercially available tests at several certified diagnostic laboratories (Quest Diagnostics, BJC Healthcare, Barnes-Jewish Hospital, Mercy Hospital, LabCorp, Missouri Baptist Medical Center).

Archived U.S. pre-COVID samples were collected at Barnes-Jewish Hospital before October 2019 (Table 1). The African pre-COVID samples were collected in western Uganda (58 samples) and in southern Cote d’Ivoire (30 samples).^18^^,^^19^ All archived plasma samples had been stored at –20°C. All samples in this study were divided into aliquots in identical plastic tubes and labeled with study-specific barcodes. The study data manager held the key linking barcodes to sample numbers and their metadata. Aliquots of samples were stored at 4°C no longer than 2 weeks prior to use.

**Table 1:** Overview of the sample panel used for the evaluation

Sample group		*P* total	*P*/ group	N samples	N samples/ group	Date collected	Location	Remarks
COVID-19	Acute cases 0–3 d	89*	31	210	32	March–May 2020	BJH, St. Louis, MO	Cases confirmed by RT-PCR at BJH
	Acute cases 4–7 d		45		50	March–May 2020		
	Acute cases 8–14 d		54		71	March–May 2020		
	Acute cases > 14 d		36		57	March–May 2020		
	Convalescent cases > 21 d	80	80	80	80	March–May 2020		Cases with reported positive RT-PCR results
Pre-COVID-19	United States	80	80	80	80	March–May 2019, 2007		Archived, de-identified samples
	Africa	88	88	88	88	Uganda: 1991 and 1995; Cote d’Ivoire, 2016	Ntoroko, western Uganda; Agboville, southeastern Cote d’Ivoire	9 of the 58 samples from Uganda were from HIV-positive individuals.^19^

BJH = Barnes-Jewish Hospital; COVID-19 = coronavirus disease 2019; N = number; P =person; RT-PCR = reverse transcription–polymerase chain reaction. COVID-19 cases were grouped by the duration of symptoms at the time when samples were collected. Plasma samples were collected using ethylenediaminetetraacetic acid as an anticoagulant.

* The same patient may have provided samples at different time points and may be represented in different groups.

### Antibody test kits.

We evaluated six rapid-format, point-of-care LFA antibody test kits that detect both IgM and IgG antibodies to recombinant viral proteins (Table 2). The kits were selected based on availability and provision of tests by the sponsor. A seventh rapid test kit (qSARS-COV-2 IgG/IgM cassette rapid test; Cellex, Research Triangle Park, NC) was evaluated, but results were excluded from this report based on requests from the manufacturer and the donor of the test kits (Foundation for Innovative New Diagnostics, Geneva, Switzerland) related to concerns about product integrity and performance of the lots tested. No results were excluded from the analysis based on requests from funders or manufacturers after results were disclosed to them.

**Table 2:** Summary of the manufacturer, production lot, diagnostic antigens, and date of emergency use authorization by the U.S. Food and Drug Administration for the tests evaluated in our study

Company		Test	Lot	Antigen	Date EUA issued (July 2020)
Lateral flow assays	BioMedomics, Inc., St. Ingbert, Germany	COVID-19 IgM-IgG Combined Antibody Rapid Test	51-200404	RBD	–
	BTNX, Inc., Markham, Ontario, Canada	Rapid Response COVID-19 IgG/IgM Test Cassette	I2003190, CI20E66 (Liberty version)	NP, S1	–
	Alfa Scientific Design Inc., Poway CA; USAAlfa	AlfaCOVID 19 Rapid Test Cassettes	PD200406C	SP, NP	–
	Innovita Biological Technology Co., Ltd, Tangshan, China	Innovita 2019-nCoV Ab Test (colloidal gold)	20200403	SP, NP	–
	SALOFA OY, Salo, Finland	Sienna/COVIBlock, COVID-19 IgG/IgM Rapid Test Cassettes	20051104	SP	07/13/2020
	VivaChek Biotech Co., Ltd., Hangzhou, China	VivaDiag^TM^ COVID-19 IgM/IgG Rapid Test	E2003002	Recombinant antigen*	–
ELISA	EUROIMMUN U.S. Inc., Lübeck, Germany	SARS-CoV-2 ELISA (IgG)	E200511BS	S1	05/04/2020
Automated CMIA	Abbott Laboratories, Inc., Lake Forest, IL	Architect SARS-CoV-2 IgG	16020M800	NP	04/26/2020

CMIA = chemiluminescent microparticle assay; COVID-19 = coronavirus disease 2019; EUA = emergency use authorization; NP = nucleocapsid protein; RBD = receptor-binding domain of the spike protein; S1 = variable domain of the spike protein; SARS-CoV-2 = severe acute respiratory syndrome coronavirus 2; SP = spike protein.

* Used antigen not disclosed.

### Test procedures.

We performed the EUROIMMUN ELISA and LFA tests according to the manufacturers’ instructions for use. The Abbott Architect chemiluminescent microparticle assay (CMIA) IgG test was performed in a clinical laboratory at Barnes-Jewish Hospital in St. Louis, MO (https://www.barnesjewish.org/Medical-Services/Laboratory-Services) according to the company’s instructions for use. Persons who performed the tests did not have access to study sample numbers, RT-PCR results, or metadata. Samples were tested in several batches and randomized within the batch. LFA tests were read by two independent readers, and results were recorded on a paper form that contained the matching barcode. Results were compared, and a third reader was used in the case of discordant results. All LFA and ELISA testing was performed with the same panel of 458 samples (Table 1). Only 413 samples were tested with the Abbott CMIA test, because this test required 200 μL plasma, and we did not have enough plasma to test some of the samples.

### Data analysis.

Antibody test results for each test kit (IgM, IgG antibody) were entered into a test result database with the participant’s unique identifier barcode number using double data entry. Results were merged into a database that contained the unique identifier barcode number, the participant identification number for the parent studies WU-350 and WU-353, and metadata. Practical characteristics related to test acceptability were scored by technicians who performed the tests using a preprinted test evaluation form. Sensitivity and specificity were calculated using standard methods, and binomial 95% CIs were calculated for these estimates. Pairwise comparison analyses of differences in test results were performed with McNemar’s test, and *P* values were adjusted using a false-discovery rate adjustment.^20^ All analyses were conducted using SAS v. 9.4 (SAS Institute, Cary, NC).

## RESULTS

### Sensitivity.

The sensitivity of the six LFAs and two laboratory-based tests varied by test and by time after the onset of COVID-19 symptoms (Table 3). The sensitivity of the LFAs to detect IgM or IgG antibodies were between 18.8% and 40.6% 0 to 3 days after the onset of symptoms, and peaked between 80.3% and 96.4% more than 14 days after the onset of symptoms (Table 3). IgM positivity rates tended to be greater than IgG rates for the first 2 weeks after symptom onset, but there was little difference at later time points. The sensitivity of the laboratory-based EUROIMMUN ELISA and the Abbott CMIA IgG tests was 15.6% and 21.4% 0 day and 1 to 3 days after the onset of symptoms, respectively, and were 89.1% and 93.1%, respectively, more than 14 days after the onset of symptoms. High sensitivity was not linked to the antigen or antigen combination used in the tests (compare Tables 2 and 3).

**Table 3:** Sensitivity for IgM and/or IgG antibodies in plasma specimens from patients with positive SARS-CoV2 reverse transcription–polymerase chain reaction by antibody test and days since onset of symptoms

Test and duration of symptoms	Total P	Total N	Detected Antibody Type								
			IgM			IgG			IgM or IgG		
			Positive	%	95% CI	Positive	%	95% CI	Positive	%	95% CI
BioMedomics											
0–3 d	31	32	6	18.8	7.2–36.4	3	9.4	2–25	6	18.8	7.2–36.4
4–7 d	45	50	20	40	26.4–54.8	14	28	16.2–42.5	23	46	31.8–60.7
8–14 d	54	71	44	62	49.7–73.2	36	50.7	38.6–62.8	50	70.4	58.4–80.7
> 14 d	116	137	96	70.1	61.7–77.6	114	83.2	75.9–89	116	84.7	77.5–90.3
BTNX											
0–3 d	31	32	13	40.6	23.7–59.4	7	21.9	9.3–40	13	40.6	23.7–59.4
4–7 d	45	50	32	64	49.2–77.1	20	40	26.4–54.8	34	68	53.3–80.5
8–14 d	54	71	61	85.9	75.6–93	46	64.8	52.5–75.8	62	87.3	77.3–94
> 14 d	116	137	130	94.9	89.8–97.9	123	89.8	83.4–94.3	132	96.4	91.7–98.8
Alfa											
0–3 d	31	32	10	31.3	16.1–50	2	6.3	0.8–20.8	10	31.3	16.1–50
4–7 d	45	50	26	52	37.4–66.3	15	30	17.9–44.6	28	56	41.3–70
8–14 d	54	71	55	77.5	66–86.5	46	64.8	52.5–75.8	57	80.3	69.1–88.8
> 14 d	116	137	113	82.5	75.1–88.4	113	82.5	75.1–88.4	124	90.5	84.3–94.9
Innovita											
0–3 d	31	32	7	21.9	9.3–40	3	9.4	2–25	7	21.9	9.3–40
4–7 d	45	50	19	38	24.7–52.8	13	26	14.6–40.3	20	40	26.4–54.8
8–14 d	54	71	48	67.6	55.5–78.2	39	54.9	42.7–66.8	49	69	56.9–79.5
> 14 d	116	137	101	73.7	65.5–80.9	96	70.1	61.7–77.6	110	80.3	72.6–86.6
Sienna											
0–3 d	31	32	9	28.1	13.7–46.7	4	12.5	3.5–29	9	28.1	13.7–46.7
4–7 d	45	50	20	40	26.4–54.8	16	32	19.5–46.7	22	44	30–58.7
8–14 d	54	71	54	76.1	64.5–85.4	38	53.5	41.3–65.5	55	77.5	66–86.5
> 14 d	116	137	124	90.5	84.3–94.9	117	85.4	78.4–90.8	129	94.2	88.8–97.4
VivaDiagTM											
0–3 d	31	32	8	25	11.5–43.4	8	25	11.5–43.4	8	25	11.5–43.4
4–7 d	45	50	22	44	30–58.7	17	34	21.2–48.8	22	44	30–58.7
8–14 d	54	71	52	73.2	61.4–83.1	50	70.4	58.4–80.7	52	73.2	61.4–83.1
> 14 d	116	137	119	86.9	80–92	118	86.1	79.2–91.4	119	86.9	80–92
EUROIMMUN ELISA											
0–3 d	31	32				5*	15.6	5.3–32.8	5*	15.6	5.3–32.8
4–7 d	45	50				14^†^	28	16.2–42.5	14^†^	28	16.2–42.5
8–14 d	54	71				44*	62	49.7–73.2	44*	62	49.7–73.2
> 14 d	116	137				122*	89.1	82.6–93.7	122*	89.1	82.6–93.7
Abbott Architect CMIA											
0–3 d	27	28				6	21.4	8.3–41	6	21.4	8.3–41
4–7 d	40	44				15	34.1	20.5–49.9	15	34.1	20.5–49.9
8–14 d	49	63				41	65.1	52–76.7	41	65.1	52–76.7
> 14 days	111	130				121	93.1	87.3–96.8	121	93.1	87.3–96.8

N = number; P =person; SARS-CoV-2 = severe acute respiratory syndrome coronavirus 2.

* One additional sample was undetermined.

† Two additional samples were undetermined.

Samples from three patients (2.6% of all patients) collected 27, 31, and 38 days after the onset of symptoms were antibody negative with all eight tests. None of these three study participants had a history of diseases or treatments known to affect immune responses to infections or vaccines. Sixteen patients provided samples at multiple time points > 14 days after onset of symptoms, and most samples were positive with all tests. One patient tested negative with two samples at days 16 and 23 with the BioMedomics test. A different patient was negative for antibodies on day 15 with five tests, but tested positive with all tests at day 18.

We performed pairwise comparisons of the sensitivity of the tests. There were no significant differences between the outcomes (IgM, IgG, and IgM or IgG) 0 to 3 days after the onset of symptoms; there were between 0 and 10 significant differences 4 to 7 days after symptom onset and between 4 and 10 significant differences in the 8- to 14-day time period. The largest differences in sensitivity occurred > 14 days after symptom onset, with 14, 13, and 11 significant differences for IgM or IgG, IgG, and IgM, respectively (Supplemental Tables S1 and S2). At that time, the BTNX and Sienna/COVIBlock tests had the greatest sensitivity estimates, with 96.4% and 94.2%, respectively. Sienna had significantly greater sensitivity compared with three of seven tests, and the BTNX LFA had significantly greater sensitivity compared with five of the seven tests (Supplemental Tables S1 and S2).

### Specificity.

Test specificity was highly variable, with a range between 82.7% (BTNX) and 95.2% to 95.8% (BioMedomics, Innovita, Sienna) (Table 4). Specificity rates tended to be greater for IgG (92.9–100%) than for IgM (83.7–95.2%). Also, test specificity was slightly greater (especially for IgM) for pre-pandemic samples from the United States than for those from Africa (Supplemental Table S3). The combined specificity of LFAs with pre-pandemic samples was 81.3% for U.S. pre-COVID-19 samples, but only 68.2% for sub-Saharan pre-COVID-19 samples (*P* = 0.053), but this difference was mostly a result of lower IgM specificity (82.5% versus 68.2%, *P* = 0.032). Pairwise test comparisons show no significant specificity differences for IgG with samples from the United States and from Africa, but differences were observed for IgM for seven test pairs with samples from the United States and 11 test pairs with samples from sub-Saharan Africa (Supplemental Table S4). These results show the importance of evaluating specificity of LFAs with samples from different geographic regions, especially if IgM test results are considered.

**Table 4:** Specificity of nine lateral flow assays and two laboratory tests to detect IgM and/or IgG antibodies in plasma specimens collected before May 2019 from individuals visiting a hospital in St. Louis, MO, or living in villages in western Uganda or southeastern Cote d’Ivoire

Test	Detected antibody type									
	IgM				IgG			IgM or IgG		
	*N*	Negative	%	95% CI	Negative (*n*)	%	95% CI	Negative (*n*)	%	95% CI
BioMedomics										
Total control	168	161	95.8	91.6–98.3	166	98.8	95.8–99.9	161	95.8	91.6–98.3
U.S. control	80	77	96.3	89.4–99.2	80	100	95.5–100	77	96.3	89.4–99.2
African control	88	84	95.5	88.8–98.7	86	97.7	92–99.7	84	95.5	88.8–98.7
BTNX										
Total control	168	141	83.9	77.5–89.1	165	98.2	94.9–99.6	139	82.7	76.2–88.1
U.S. control	80	70	87.5	78.2–93.8	79	98.8	93.2–100	69	86.3	76.7–92.9
African control	88	71	80.7	70.9–88.3	86	97.7	92–99.7	70	79.5	69.6–87.4
Alfa										
Total control	168	161	95.8	91.6–98.3	166	98.8	95.8–99.9	159	94.6	90.1–97.5
U.S. control	80	76	95	87.7–98.6	80	100	95.5–100	76	95	87.7–98.6
African control	88	85	96.6	90.4–99.3	86	97.7	92–99.7	83	94.3	87.2–98.1
Innovita										
Total control	168	161	95.8	91.6–98.3	167	99.4	96.7–100	160	95.2	90.8–97.9
U.S. control	80	78	97.5	91.3–99.7	80	100	95.5–100	78	97.5	91.3–99.7
African control	88	83	94.3	87.2–98.1	87	98.9	93.8–100	82	93.2	85.7–97.5
Sienna										
Total control	168	160	95.2	90.8–97.9	168	100	97.8–100	160	95.2	90.8–97.9
U.S. control	80	79	98.8	93.2–100	80	100	95.5–100	79	98.8	93.2–100
African control	88	81	92	84.3–96.7	88	100	95.9–100	81	92	84.3–96.7
VivaDiagTM										
Total control	168	156	92.9	87.9–96.3	162	96.4	92.4–98.7	156	92.9	87.9–96.3
U.S. control	80	77	96.3	89.4–99.2	78	97.5	91.3–99.7	77	96.3	89.4–99.2
African control	88	79	89.8	81.5–95.2	84	95.5	88.8–98.7	79	89.8	81.5–95.2
Any lateral flow assay										
Total control	168	126	75	67.7–81.3	158	94	89.3–97.1	125	74.4	67.1–80.8
U.S. control	80	66	82.5	72.4–90.1	77	96.3	89.4–99.2	65	81.3	71–89.1
African control	88	60	68.2	57.4–77.7	81	92	84.3–96.7	60	68.2	57.4–77.7
EUROIMMUN ELISA										
Total control	168				163*	97	93.2–99	163*	97	93.2–99
U.S. control	80				79^†^	98.8	93.2–100	79^†^	98.8	93.2–100
African control	88				84^‡^	95.5	88.8–98.7	84^‡^	95.5	88.8–98.7
Abbott Architect CMIA										
Total control	156				147	94.2	89.3–97.3	147	94.2	89.3–97.3
U.S. control	69				69	100	94.8–100	69	100	94.8–100
African control	87				78	89.7	81.3–95.2	78	89.7	81.3–95.2

* Included seven indeterminate samples.

† Included four indeterminate samples.

‡ Included three indeterminate samples.

### Effects of requiring two different positive tests on sensitivity and specificity.

We considered the effect of requiring positive test results for two different LFAs as a criterion for calling a sample antibody positive. First, we compared the agreement of test results from the 290 samples from COVID-19-positive subjects. The agreement of positive results (IgM or IgG) between different LFAs were between 80.6% (BTNX and Innovita) and 92.5% (Sienna and Alfa) (Supplemental Table S5). Agreement results for IgM alone were between 75.4% (BTNX and BioMedomics) and 89.4% (Sienna and Alfa), and for IgG were between 78.6% (BTNX and Innovita) and 94.5% (Sienna and BioMedomics). Next, we considered results when one LFA was used as a screening test and a second LFA was used as a confirmatory test (Table 5). Both tests had to be positive (for either IgM or IgG) for a sample to be considered positive for antibodies. All pairs of LFAs were considered. For LFAs, three combinations had specificities greater than 99%: BioMedomics/Sienna (99.4%), Alfa/Sienna (99.4%), and BioMedomics/VivaDiag^TM^ (100%). These combinations had sensitivities for COVID-19 samples (> 14 days) of 80.3% for BioMedomics/VivaDiag^TM^, 83.9% for BioMedomics/Sienna and 89.1% for Alfa/Sienna. Thus, the best LFA combination was Alfa/Sienna when both sensitivity and specificity are considered. We next considered combinations of one LFA with either ELISA or CMIA. All the LFAs had > 98.5% specificity when a positive ELISA or CMIA was required as confirmation for a positive overall result. BioMedomics, Innovita, and Sienna had 100% specificity when they were combined with results from either ELISA or CMIA. Requiring confirmatory results by ELISA or CMIA reduced sensitivities for these LFAs from between 80.3% and 94.2% to between 76.6% and 90%. Thus, these results show that combined use of two LFAs or an LFA with ELISA or CMIA confirmatory test increased specificity to > 99% without a major reduction in sensitivity.

**Table 5:** Specificity (for pre-coronavirus disease 2019 samples) and sensitivity (for confirmed pre-coronavirus disease 2019 sample > 14 days after onset of symptoms) of lateral flow assays if two different tests are combined, with a screening tests and a confirmatory test for IgM or IgG positive samples

Screening test	Confirmation test	Specificity (95% CI)	Sensitivity (95% CI)
BioMedomics	BTNX	95.8% (91.6–98.3)	83.9% (76.7–89.7)
	Alfa	97.6% (94–99.3)	83.9% (76.7–89.7)
	Innovita	98.8% (95.8–99.9)	75.2% (67.1–82.2)
	Sienna	99.4% (96.7–100)	83.9% (76.7–89.7)
	VivaDiag^TM^	100% (97.8–100)	80.3% (72.6–86.6)
	EUROIMMUN	100% (97.8–100)	81.8% (74.3–87.8)
	Abbott	100% (97.7–100)	82.3% (74.6–88.4)
BTNX	BioMedomics	95.8% (91.6–98.3)	83.9% (76.7–89.7)
	Alfa	96.4% (92.4–98.7)	89.8% (83.4–94.3)
	Innovita	97% (93.2–99)	79.6% (71.8–86)
	Sienna	97% (93.2–99)	94.2% (88.8–97.4)
	VivaDiag^TM^	98.2% (94.9–99.6)	86.9% (80–92)
	EUROIMMUN	98.8% (95.8–99.9)	89.1% (82.6–93.7)
	Abbott	99.4% (96.5–100)	92.3% (86.3–96.2)
Alfa	BioMedomics	97.6% (94–99.3)	83.9% (76.7–89.7)
	BTNX	96.4% (92.4–98.7)	89.8% (83.4–94.3)
	Innovita	98.8% (95.8–99.9)	78.8% (71–85.3)
	Sienna	99.4% (96.7–100)	89.1% (82.6–93.7)
	VivaDiag^TM^	98.8% (95.8–99.9)	83.2% (75.9–89)
	EUROIMMUN	99.4% (96.7–100)	83.9% (76.7–89.7)
	Abbott	100% (97.7–100)	86.2% (79–91.6)
Innovita	BioMedomics	98.8% (95.8–99.9)	75.2% (67.1–82.2)
	BTNX	97% (93.2–99)	79.6% (71.8–86)
	Alfa	98.8% (95.8–99.9)	78.8% (71–85.3)
	Sienna	98.8% (95.8–99.9)	78.8% (71–85.3)
	VivaDiag^TM^	98.2% (94.9–99.6)	78.1% (70.2–84.7)
	EUROIMMUN	100% (97.8–100)	76.6% (68.7–83.4)
	Abbott	100% (97.7–100)	78.5% (70.4–85.2)
Sienna	BioMedomics	99.4% (96.7–100)	83.9% (76.7–89.7)
	BTNX	97% (93.2–99)	94.2% (88.8–97.4)
	Alfa	99.4% (96.7–100)	89.1% (82.6–93.7)
	Innovita	98.8% (95.8–99.9)	78.8% (71–85.3)
	VivaDiag^TM^	100% (97.8–100)	84.7% (77.5–90.3)
	EUROIMMUN	100% (97.8–100)	87.6% (80.9–92.6)
	Abbott	100% (97.7–100)	90% (83.5–94.6)
VivaDiag^TM^	BioMedomics	100% (97.8–100)	80.3% (72.6–86.6)
	BTNX	98.2% (94.9–99.6)	86.9% (80–92)
	Alfa	98.8% (95.8–99.9)	83.2% (75.9–89)
	Innovita	98.2% (94.9–99.6)	78.1% (70.2–84.7)
	Sienna	100% (97.8–100)	84.7% (77.5–90.3)
	EUROIMMUN	99.4% (96.7–100)	83.2% (75.9–89)
	Abbott	99.4% (96.5–100)	86.2% (79–91.6)
EUROIMMUN ELISA	BioMedomics	100% (97.8–100)	81.8% (74.3–87.8)
	BTNX	98.8% (95.8–99.9)	89.1% (82.6–93.7)
	Alfa	99.4% (96.7–100)	83.9% (76.7–89.7)
	Innovita	100% (97.8–100)	76.6% (68.7–83.4)
	Sienna	100% (97.8–100)	87.6% (80.9–92.6)
	VivaDiag^TM^	99.4% (96.7–100)	83.2% (75.9–89)
	Abbott	99.4% (96.5–100)	87.7% (80.8–92.8)
Abbott Architect CMIA	BioMedomics	100% (97.7–100)	82.3% (74.6–88.4)
	BTNX	99.4% (96.5–100)	92.3% (86.3–96.2)
	Alfa	100% (97.7–100)	86.2% (79–91.6)
	Innovita	100% (97.7–100)	78.5% (70.4–85.2)
	Sienna	100% (97.7–100)	90% (83.5–94.6)
	VivaDiag^TM^	99.4% (96.5–100)	86.2% (79–91.6)
	EUROIMMUN	99.4% (96.5–100)	87.7% (80.8–92.8)

Samples that were positive with the first test, but negative with the confirmatory were considered indeterminate/negative.

**Table 6:** Sensitivity and specificity to detect IgM or IgG of the tests evaluated in our study compared with results from previous studies

Company		% Sensitivity, > 14days after onset of COVID-19 symptoms	Specificity (%)	Remarks	Reference
Lateral flow assays	BioMedomics, Inc.	84.7 81.4 92.7	95.8 86.9 95.9	d > 16 + d > 20	Our study, Pickering et al.,^24^ Whitman et al.^16^
	BTNX, Inc.	96.4	82.7		Our study
	Alfa	90.5 93.0	94.7 100.0		Our study, Alfa Diagnostic^25^
	Innovita Biological Technology Co., Ltd.	80.3 73.8 85.7	95.2 96.3 100	d > 16 + d > 20	Our study, Whitman et al.,^16^ Herroelen et al.^26^
	Sienna	94.2 93.3	95.2 98.8	EUA07/13/2020	Our study, U.S. Food and Drug Administration^27^
	VivaDiag^TM^	86.9 80.7 94.7	92.9 99.1 99.0	d > 16 + d > 20	Our study, Whitman et al.,^16^ Van Elslande et al.^15^
ELISA	EUROIMMUN U.S. Inc.	89.1 89.5 90.0 90.5 90.8	97.0 96.1 100.0 98.2 100		Our study, Van Elslande et al.,^15^ U.S. Food and Drug Administration,^27^ Herroelen et al.,^26^ Pickering et al.^24^
Automated chemiluminescent microparticle assay	Abbott Laboratories, Inc.	93.1 83.1 100.0	94.2 99.2 99.6		Our study, Perkmann et al.,^28^U.S. Food and Drug Administration^27^

COVID = coronavirus disease 2019.

### A comparison of two test versions from the same manufacturer.

The manufacturer of the BTNX test provided a small number of kits from a second test (the Liberty test) (Table 2) for evaluation after our main testing had been completed. We tested this test with a subset of 74 samples from subjects with COVID-19 at various time points after the onset of symptoms and 74 sample collected before the pandemic (Supplemental Table S6). Sensitivities for IgM or IgG antibodies in the sample subset were 62.2% and 64.9% for the first BTNX test, and 60.8% and 62.2% for the BTNX Liberty test, respectively. However, the numbers of positive IgM or IgG tests for pre-COVID-19 samples were 27 and 29 for the first BTNX test, but only five and six for the Liberty test, respectively. These results suggest the BTNX Liberty test had similar sensitivity but improved specificity compared with the first BTNX test.

### Test performance characteristics.

All six LFAs were easy to perform, but there were small differences related to packaging, the amount of plasma needed, incubation time, and difficulty in reading the test (Supplemental Table S7). For example, the BioMedomics test produced control lines with inconsistent intensity and more diffuse positive test lines than other tests.

## DISCUSSION

We evaluated six LFAs and two laboratory-based tests independently for detecting antibodies to SARS-CoV-2 proteins. Our results show that these tests have low sensitivity in the first week after the onset of symptoms. Sensitivity rates were much greater for samples collected more than 14 days after symptom onset. These results are consistent with results from previous studies and a number of meta-analyses that evaluated some of the same tests (Table 6).^15^^,^^21^^,^^22^ This means that although LFAs have relatively limited value for diagnosing COVID-19 shortly after symptom onset, they have good sensitivity later during the course of the infection. Because antigen detection LFAs are readily available, the main use case for antibody LFAs is for detecting anti-spike protein antibodies after natural infection or vaccination. Because currently available vaccines induce antibodies against the spike protein, only tests that detect antibodies against that protein are suitable for assessing responses to vaccines.

We compared the sensitivity of the six LFAs and two laboratory-based tests evaluated in our study with data from other independent evaluation studies or with data provided by the manufacturer (Table 6). This analysis focused on sensitivity for samples from subjects collected more than 14 days after the onset of symptoms. The most extensive data were available for the EUROIMMUN ELISA. The sensitivity of the Abbott CMIA has been reported to be between 83% and 100%, and it was 91.8% in our study. Sensitivities for the LFAs in our study were in the same range as in previous studies (80–95%). Specificities in our study were also generally similar to those noted in previous reports. However, we did not confirm exceptionally high specificities of 99% to 100%, which have been reported for some of the tests we evaluated.^14^ This may be a result, in part, of the fact that our study included pre-COVID-19 samples from sub-Saharan Africa.

Specificity is a challenge for SARS-CoV-2 antibody tests. This is especially true for LFAs when looking at IgM. Although IgM appears earlier than IgG after the onset of symptoms (Table 3), we found that the specificity is lower compared with IgG, and IgM results alone add little reliable information. However, specificity of LFAs could be increased to > 99% by requiring positive test results with certain two-test combinations with only minor reductions in sensitivity. In addition, our results suggest that the best LFAs had similar sensitivities and specificities as the two laboratory-based antibody tests (ELISA and CMIA). Thus, LFAs may be a good alternative to expensive and technically demanding laboratory-based tests. This is especially true for settings in which immediate results for individual samples are desired and in low-resource settings in the developing world. On the other hand, automated tests may be preferable for mass testing in high-resource settings,

The WHO developed a target product profile for rapid antibody tests.^23^ According to this profile, rapid point-of-care tests to detect prior infection should have a minimal sensitivity of 90% and a minimal specificity of 97%. Our results show that none of the tests we evaluated satisfied these targets. However, certain two-test combinations satisfied the specificity target and provided good sensitivity for samples collected more than 14 days after the onset of symptoms

All the tests we evaluated were newly developed and the in early stages of commercialization at the time of our study. We originally evaluated seven LFAs, but results from the Cellex test were redacted from the study because of logistic problems and shipping delays that might have compromised test performance. Furthermore, the BioMedomics and BTNX tests we evaluated were recalled 8 and 10 weeks after we evaluated these tests, respectively. The companies thought that delays during transit may have affected test performance. However, we included the results from these tests because no obvious problems were noted during our evaluation. We think it is unlikely that shipping delays would decrease test specificity. The BTNX Liberty test that we evaluated with a subset of samples had greater specificity than the original BTNX test, and this may justify a more thorough evaluation. The BioMedomics test produced a rather diffuse positive test band compared with those in other tests. Four of the six LFAs produced inconsistent intensities of control lines. Although this is not important for qualitative detection, it may be a problem for semiquantitative or quantitative detection if the control line is used for comparison. The companies may be able fix this in future versions of the tests. These experiences and others illustrate problems that can occur when tests are moved rapidly to the market and when shipments are delayed because of shipping and customs clearance.

Although we do not know the retail costs for the LFAs that we evaluated, LFAs are generally less expensive than laboratory-based assays when equipment and personnel costs are included in the cost analysis. The LFAs work with very small sample volumes and with a variety of specimens (plasma, serum, whole blood). They enable rapid point-of-care testing for antibodies with capillary blood samples. As alluded to earlier, point-of-care testing may be especially useful in low- and middle-income countries where transport of specimens to centralized testing facilities and delays in reporting results are often major challenges.

A recent systematic review and meta-analysis concluded that the available evidence does not support the continued use of existing point-of-care antibody tests.^21^ Although this may be true for persons with early infections, we think these tests have real value for certain use cases. Another systematic review concluded the sensitivity of antibody tests is too low in the first week after symptom onset to have a primary role in the early diagnosis of COVID-19, but they may still have a role for documenting recent infections in individuals later in the course of their illness, when RT-PCR tests are either missing or negative. The same study acknowledged that antibody tests are likely to have a role for documenting previous SARS-CoV-2 infections with samples that are collected 15 or more days after the onset of symptoms.^22^ Our study supports this view, with additional data obtained with six LFAs and a large panel of well-characterized plasma samples.

Our study has a few limitations. We focused on plasma samples that were collected in ethylenediaminetetraacetic acid-coated vacutainers and were stored/handled optimally, which might not always be the case in remote settings where rapid LFAs could be especially valuable. The positive samples we tested were from persons with clinical symptoms who presented to a hospital or testing station. Thus, we have no data on the performance of these tests with samples from persons with asymptomatic infections. Another limitation of our study is that we no data on the duration of antibody responses after SARS-CoV-2 infection. We also do not know whether antibodies detected by LFAs correlate with protective immunity or whether these tests might be useful for assessing immune responses after vaccination. These are important questions that merit further study.

In conclusion, our study shows that a subset of the LFAs that we examined had comparable sensitivities and specificities to laboratory-based ELISA or CMIA tests for antibodies to SARS-CoV-2. Sensitivities for active or recent infection increased with time after the onset of symptoms, and these values were very good 14 days or longer after symptom onset. The best tests we evaluated had good specificity, but there is room for improvement. Dual testing with certain test combinations provided excellent specificity. Thus, we believe that currently available LFAs provide clinically useful information regarding current or recent COVID-19 in individuals. Additional studies should be performed to assess their value as surveillance tools for SARS-CoV-2 in populations and for documenting antibody responses to vaccines.

## References

[1] John Hopkins University , 2020. *Coronavirus Resource Center*. Available at: https://coronavirus.jhu.edu/map.html. Accessed October 13, 2020.

[2] Corman VM 2020. Detection of 2019 novel coronavirus (2019-nCoV) by real-time RT-PCR. Euro Surveill 25: 2000045. 10.2807/1560-7917.ES.2020.25.3.2000045 PMC6988269 31992387

[3] Tang YW Schmitz JE Persing DH Stratton CW , 2020. The laboratory diagnosis of COVID-19 infection: current issues and challenges. J Clin Microbiol 58: e00512–e00520. 32245835 10.1128/JCM.00512-20 PMC7269383

[4] Li H Pan J Su Y Wang B Ge J , 2020. SARS-CoV-2 IgM/IgG antibody detection confirms the infection after three negative nucleic acid detection. J Cell Mol Med 24: 8262–8265. 32431052 10.1111/jcmm.15275 PMC7280606

[5] Sethuraman N Jeremiah SS Ryo A , 2020. Interpreting diagnostic tests for SARS-CoV-2. JAMA 323: 2249–2251. 32374370 10.1001/jama.2020.8259

[6] Corman VM Rabenau HF Adams O Oberle D Funk MB Keller-Stanislawski B Timm J Drosten C Ciesek S , 2020. SARS-CoV-2 asymptomatic and symptomatic patients and risk for transfusion transmission. Transfusion 60: 1119–1122. 32361996 10.1111/trf.15841 PMC7267331

[7] Burbelo PD Riedo FX Morishima C Rawlings S Smith D Das S Strich JR Chertow DS Davey RT Cohen JI , 2020. Sensitivity in detection of antibodies to nucleocapsid and spike proteins of severe acute respiratory syndrome coronavirus 2 in patients with coronavirus disease 2019. J Infect Dis 222: 206–213. 32427334 10.1093/infdis/jiaa273 PMC7313936

[8] Demey B Daher N Francois C Lanoix JP Duverlie G Castelain S Brochot E , 2020. Dynamic profile for the detection of anti-SARS-CoV-2 antibodies using four immunochromatographic assays. J Infect 81: e6–e10. 10.1016/j.jinf.2020.04.033 PMC7204722 32389784

[9] Hoffman T Nissen K Krambrich J Ronnberg B Akaberi D Esmaeilzadeh M Salaneck E Lindahl J Lundkvist A , 2020. Evaluation of a COVID-19 IgM and IgG rapid test: an efficient tool for assessment of past exposure to SARS-CoV-2. Infect Ecol Epidemiol 10: 1754538. 32363011 10.1080/20008686.2020.1754538 PMC7178815

[10] Li Z 2020. Development and clinical application of a rapid IgM-IgG combined antibody test for SARS-CoV-2 infection diagnosis. J Med Virol 92: 1518–1524. 32104917 10.1002/jmv.25727 PMC7228300

[11] Montesinos I 2020. Evaluation of two automated and three rapid lateral flow immunoassays for the detection of anti-SARS-CoV-2 antibodies. J Clin Virol 128: 104413. 32403010 10.1016/j.jcv.2020.104413 PMC7198434

[12] Okba NMA 2020. Severe acute respiratory syndrome coronavirus 2-specific antibody responses in coronavirus disease 2019 patients. Emerg Infect Dis 26: 1478–1488. 32267220 10.3201/eid2607.200841 PMC7323511

[13] Foundation for Innovative New Diagnostics , 2020. *FIND Evaluation Update: SARS-COV-2 Immunoassays*. Available at: https://www.finddx.org/covid-19/sarscov2-eval-immuno/. Accessed July 20, 2020.

[14] United States Food and Drug Administration. Available at: https://www.fda.gov/medical-devices/coronavirus-disease-2019-covid-19-emergency-use-authorizations-medical-devices/eua-authorized-serology-test-performance. Accessed October 1, 2020.

[15] Van Elslande J Houben E Depypere M Brackenier A Desmet S Andre E Van Ranst M Lagrou K Vermeersch P , 2020. Diagnostic performance of 7 rapid IgG/IgM antibody tests and the EUROIMMUN IgA/IgG ELISA in COVID-19 patients. Clin Microbiol Infect 26: 1082–1087. 32473953 10.1016/j.cmi.2020.05.023 PMC7255746

[16] Whitman JD 2020. Evaluation of SARS-CoV-2 serology assays reveals a range of test performance. Nat Biotechnol 38: 1174–1183. 32855547 10.1038/s41587-020-0659-0 PMC7740072

[17] Adams ER , 2020. Antibody testing for COVID-19: a report from the National COVID Scientific Advisory Panel. Wellcome Open Res 5: 139. 33748431 10.12688/wellcomeopenres.15927.1 PMC7941096

[18] Andersen BJ 2019. Systems analysis-based assessment of post-treatment adverse events in lymphatic filariasis. PLoS Negl Trop Dis 13: e0007697. 31557154 10.1371/journal.pntd.0007697 PMC6762072

[19] Kipp W Kabwa P Verbeck A Fischer P Eggert P Buttner DW , 1995. Prevalence and risk factors of HIV-1 infection in three parishes in western Uganda. Trop Med Parasitol 46: 141–146. 8533014

[20] Benjamini Y Hochberg Y , 1995. Controlling the false discovery rate: a practical and powerful approach to multiple testing. J R Stat Soc B 57: 289–300.

[21] Bastos ML 2020. Diagnostic accuracy of serological tests for COVID-19: systematic review and meta-analysis. BMJ 370: m2515. 10.1136/bmj.m2516 PMC7327913 32611558

[22] Deeks JJ 2020. Antibody tests for identification of current and past infection with SARS-CoV-2. Cochrane Database Syst Rev 6: CD013652. 32584464 10.1002/14651858.CD013652 PMC7387103

[23] World Health Organization , 2020. Target Product Profile for Priority Diagnostics to Support Response to the COVID-19 Pandemic, v.10. Geneva, Switzerland: WHO.

[24] Pickering S 2020. Comparative assessment of multiple COVID-19 serological technologies supports continued evaluation of point-of-care lateral flow assays in hospital and community healthcare settings. PLoS Pathog 16: e1008817. 32970782 10.1371/journal.ppat.1008817 PMC7514033

[25] Alfa , 2020. *Alfa Coviblock COVID-19 Rapid Test Cassettes*. Available at: https://www.alfascientific.com/covid-19/. Accessed October 13, 2020.

[26] Herroelen PH Martens GA De Smet D Swaerts K Decavele AS , 2020. Humoral immune response to SARS-CoV-2. Am J Clin Pathol 154: 610–619. 32808976 10.1093/ajcp/aqaa140 PMC7454302

[27] U.S. Food and Drug Administration , 2020. *COVID-19: In Vitro Diagnostics EUAs.* Available at: https://www.fda.gov/medical-devices/coronavirus-disease-2019-covid-19-emergency-use-authorizations-medical-devices/vitro-diagnostics-euas#individual-serological. Accessed October 13, 2020.

[28] Perkmann T 2020. Side by side comparison of three fully automated SARS-CoV-2 antibody assays with a focus on specificity. Clin Chem 66: 1405–1413. 32777031 10.1093/clinchem/hvaa198 PMC7454460

